# Wheel Out-of-Roundness Detection Using an Envelope Spectrum Analysis

**DOI:** 10.3390/s23042138

**Published:** 2023-02-14

**Authors:** Vítor Gonçalves, Araliya Mosleh, Cecília Vale, Pedro Aires Montenegro

**Affiliations:** CONSTRUCT—LESE, Faculty of Engineering, University of Porto, 4200-465 Porto, Portugal

**Keywords:** algorithm, numerical simulation, train–track interaction, validation, wheel out-of-roundness, wayside condition monitoring, wheel flat, polygonised wheel

## Abstract

This paper aims to detect railway vehicle wheel flats and polygonized wheels using an envelope spectrum analysis. First, a brief explanation of railway vehicle wheel problems is presented, focusing particularly on wheel flats and polygonal wheels. Then, three types of wheel flat profiles and three periodic out-of-roundness (OOR) harmonic order ranges for the polygonal wheels are evaluated in the simulations, along with analyses implemented using only healthy wheels for comparison. Moreover, the simulation implements track irregularity profiles modelled based on the US Federal Railroad Administration (FRA). From the numerical calculations, the dynamic responses of several strain gauges (SGs) and accelerometer sensors located on the rail between sleepers are evaluated. Regarding defective wheels, only the right wheel of the first wheelset is considered as a defective wheel, but the detection methodology works for various damaged wheels located in any position. The results from the application of the methodology show that the envelope spectrum analysis successfully distinguishes a healthy wheel from a defective one.

## 1. Introduction

In recent years, with the development of rail transportation and increasing demand, the weight of axle loads has gradually increased for rail networks around the world. Along with improving public mobility and helping to reduce environmental issues, the increased use of rail transportation has exposed train wheels to more demanding conditions during operation. In addition to disrupting railway operations, defective wheels can cause severe damage to infrastructures, particularly tracks, increase maintenance costs, and, if not addressed at the time, may lead to derailments. Therefore, the railway operator may propose improved maintenance programs for early detection of possible defects, leading to cost savings in the long run. Furthermore, early detection of damaged wheels is recommended in order to maintain safety and stability. Studies have shown that good contact between the wheels and the rails play a major role in vehicle-overhead system interaction [1,2]. Railway administrations take the appropriate steps to address this problem and ensure safeguards and repairs are in place. Most passenger trains now have advanced anti-slip technologies that minimize wheel–rail slippage [3] however, it cannot completely prevent wheel defects. Most wheel defect detection methods developed over the last decades can be categorized into on-board monitoring systems [4,5] and wayside monitoring systems [6,7]. On-board monitoring systems mainly rely on vibration, acoustic, image detection, and ultrasonic technologies [8,9,10,11]. These detection systems are employed primarily for monitoring track conditions rather than wheel conditions. An important issue with on-board monitoring techniques is the need for sensors installed on all wheels to monitor wheel defects effectively. This turns out to be impractical as it raises costs and comes with maintenance issues. For these reasons, the preferred method of wheel defect detection is a wayside monitoring system, in which the conditions of all wheels can be analyzed during the passage of trains [12,13,14,15,16,17,18,19]. The two primary categories of wheel tread irregularities are: (i) a localized defect in the wheel tread, such as wheel flats, spalling, and shelling, and (ii) a defect around the complete wheel circumference [20]. In this paper, we will deal with one type of defect of each group: wheel flats and polygonization.

Several researchers have studied wheel defect detection and evaluated wheel conditions during operation [21,22,23,24,25,26,27,28,29,30], including the previous works of the authors [31,32,33]. Polygonal wheel mechanics have been studied in the last few decades [34,35,36,37,38,39,40,41,42]. It is a complex subject that still requires further investigation to understand several aspects related to wheel polygonization mechanisms and their impact on train–track systems [43]. Table 1 and Table 2 list several previously developed wheel flat and polygonal wheel detection methods as well as the results of those works.

There are several research studies regarding the development of wheel condition monitoring technologies [31,32,49,50]; however, to the knowledge of the authors, detecting defective wheels with envelope spectrum analysis has been limited so far. In this research study, an envelope spectrum analysis is utilized to distinguish a defective wheel from a healthy one. Two types of wheel defect are considered: a wheel flat as a local defect and polygonization as a defect around the complete wheel circumference. One of the innovations of this research compared to the authors’ previous studies [31,32,33,44,49] is to present that the envelope spectrum method is not only capable of detecting defects with high impact frequency (wheel flat), but also that this technique is effective for detecting a defective wheel with tiny defects around the wheel (polygonization). Additionally, the effect of track irregularity, train speed, and the severity of defect in detecting the damaged wheel has been investigated.

## 2. Methodology for Defect Detection

**Figure 1:**
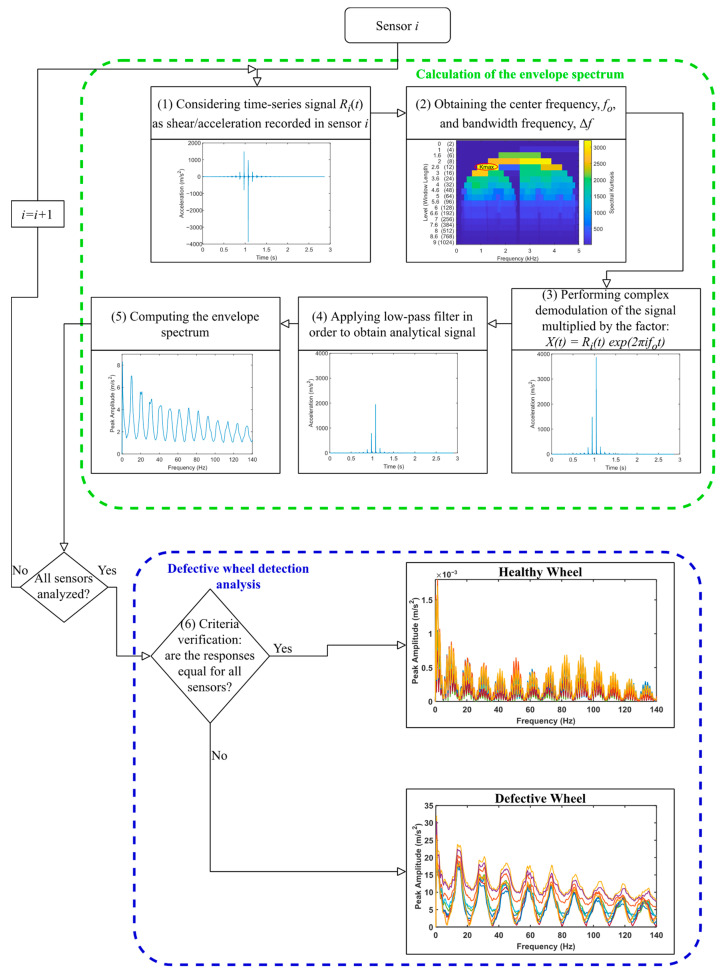
Diagram of the methodology implemented for wheel defect detection.

In this research study, envelope spectrum analysis is utilized to detect a defective wheel. Envelope spectrum analysis is a complex demodulation process implemented by shifting each frequency to zero and then applying a low-pass filter [51]. To perform a complex signal deformation, the signal is multiplied by the factor seen in Equation (1):

(1) $$X_{i}\left(t\right)=R_{i}\left(t\right)exp\left(2\pi i\;f_{0}t\right)$$

in which $f_{0}$ is the centre frequency of the band and $R_{i}\left(t\right)$ is the time-series signal. After the frequency band with the highest kurtosis level is identified, a passband filter is applied to the raw signal to obtain a higher impulse signal to analyze the envelope spectrum. Figure 1 illustrates the procedure for detecting a damaged wheel. The process included two main blocks. The first block of the flowchart, in green, refers to the calculation of the envelope spectrum. For each sensor, a time-series signal is recorded, as shear or acceleration. With this, the center frequency, $f_{0}$, and bandwidth frequency, Δf, are determined. The signal is then demodulated using Equation (1), and by applying a low-pass filter, the envelope spectrum can be calculated.

After repeating this process for all the sensors, the defective wheel detection is then performed in the second block, marked in blue. A damaged wheel is identified based on the following two criteria: (a) the lag between the signals for the damaged wheel, or (b) the amplitude difference of envelope spectrum analysis for the damaged wheel and the healthy one. If the responses from all sensors are coincident, it can be identified that the passing wheel is healthy. However, in the presence of a damaged wheel, a significant lag between amplitudes of the envelope spectrum is presented. Another indicator for detecting a defective wheel is the amplitude differences between a healthy and defective one. The amplitude of the envelope spectrum analysis for a healthy wheel is much lower than for a defective one.

Demodulation band selection for the envelope analysis of a defective wheel is often carried out by comparing the spectrum of a healthy wheel to select the particular frequencies where the greatest change occurred due to the fault. It is found that spectral kurtosis (SK) gives a very similar indication of the band being demodulated without the need for historical data. The spectral kurtosis of a signal is obtained by dividing the main signal into different frequency bands and obtaining the kurtosis of each frequency band [52]. This shows how the peak of the signal changes with frequency and can be utilized to identify the frequency band where the signal has the most impulsive behavior. This information is required to select the optimal frequency band for envelope analysis on the recorded signals.

Equations (2) and (3) [52,53] are used to obtain the bandwidth frequency and the center frequency to calculate envelope spectrum analysis:

(2) $$\Delta f=2^{\left(-l-1\right)}F_{s}$$

(3) $$f_{0}=\left(i+0.5\right)\Delta f$$

in which Δf is the bandwidth frequency; l is the level of the coefficient series; $F_{s}$ is the sampling frequency (10,000 Hz in this research study); $f_{0}$ is the center frequency; and *i* is the number of filters applied at level l. More details regarding the proposed methodology can be found in the recent publication by the authors [31,32,49].

## 3. Numerical Modelling

### 3.1. Description of the Wayside Condition Monitoring System

**Figure 2:**
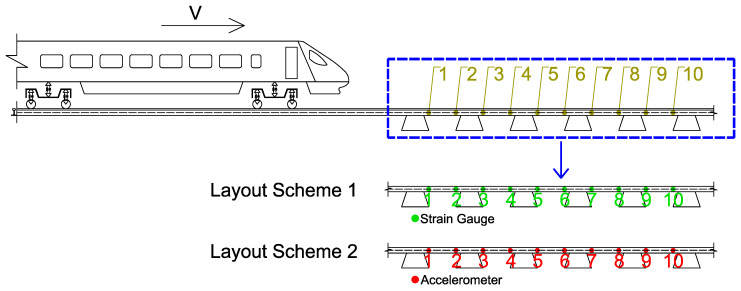
Multisensor layout schemes. Location of the sensors on the track inside the blue dotted box.

A wayside condition monitoring system consists of rail-mounted accelerometers and strain gauges (SGs). The ten positions of the track shown in Figure 2 represent the locations where the sensors would be installed in a real wayside system. In this study, two different sensor layouts, indicated in Figure 2, are considered. Sensors 1 to 10 are represented by the SGs (layout 1), or accelerometers (layout 2) located on the rail between two sleepers. Both strain gauges and accelerometers are located at the same position in order to compare their track response evaluated by different sensors.

### 3.2. Wheel Flat Profile

The wheel flat is mainly characterized by the wheel flat length (L) and the flat depth (D), calculated according to Equation (4) [54]:

(4) $$D=\frac{L^{2}}{16r_{w}}$$

in which $r_{w}$ is the radius of the wheel. In this study, the wheel flat vertical profile deviation (Z) is defined as:

(5) $$Z=-\frac{D}{2}\left(1-cos\frac{2\pi x}{L}\right)H\left(x-\left(2\pi r_{w}-L\right)\right),\;\;0\leq x\leq 2\pi r_{w}$$

in which H is the Heaviside function. When a defective wheel rotates, the flat of the wheel causes a periodic impulse to the track with a particular frequency. The frequency of the periodic impulse corresponding to the flat impact frequency ($f_{f}$) can be determined as follows:

(6) $$f_{f}=\frac{V}{\left(2\pi r_{w}\right)}$$

in which V is the train speed.

Three wheel flat profiles are analyzed in this study, along with a healthy wheel profile. The values of *L* and *D*, for the three wheel flats, are presented in Table 3.

### 3.3. Polygonized Wheel Profile

In order to replicate the effect of wheel polygonization, the circumferential irregularity for the wheel has to be modelled. Periodic out-of-roundness (OOR) is single or multiple deviation of the wheel diameter over the entire circumference of the wheel. The wavelengths *λ_θ_* correspond to the first 20 harmonic orders of a polygonized wheel is defined as:

(7) $$\lambda_{\theta }=\frac{2\pi r_{w}}{\theta },\;\left(\theta =1,\;2,\;3,\;\ldots ,\;20\right)$$

in which θ, is the harmonic order. Depending on the train speed, the OOR will cause a simultaneous wheel–rail excitation in a specific frequency range.

The initial irregularity profile is modelled as a sum of sine functions (M = 20):

(8) $$\lambda_{\theta }=\frac{2\pi r_{w}}{\theta },\;\left(\theta =1,\;2,\;3,\;\ldots ,\;20\right)$$

The amplitude of the sine function for each wavelength is obtained as:

(9) $$a_{\theta }=\sqrt{2}*10^{\frac{L_{w\theta }}{20}}*w_{ref}$$

where $w_{ref}=1$ μm, *L*_*wθ*_ can be calculated by the following function:

(10) $$L_{w\theta }=24.7\log_{10}\left(\lambda_{\theta }\right)+8.47$$

Different wheel irregularities defined by the same spectrum are generated by assuming phase angles to the sine functions that are uniformly and randomly distributed between 0 and 2π. The generated polygonization is then applied to the elements defining the geometry of the wheel surface. To develop the polygonized wheel profiles analyzed, three different OOR harmonic orders are considered for the analyses, as seen in Table 4.

### 3.4. Rail Unevenness Profile

**Figure 3:**
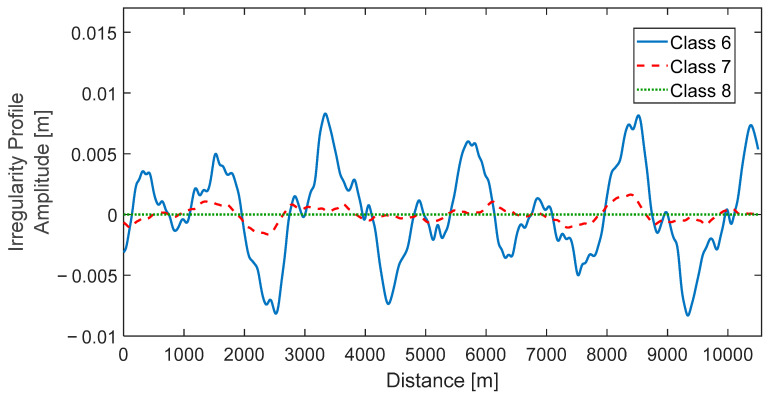
Track irregularity profiles for “classes 6 to 8”.

Since actual measurements from condition monitoring systems in Portugal are confidential, to validate the methodology, measurements are artificially created based on the real conditions in the Portuguese Railway Northern Line. Since real railway tracks are not completely perfect, track irregularities affect the dynamic response of the track and, therefore, must be considered in the analysis. To simulate the artificial track unevenness profiles, a stationary stochastic process characterized by a power spectral density (PSD) function is used according to Equation (11) [56,57].

(11) $$S\left(K_{1}\right)=\frac{10^{-7}AK_{3}^{2}\left(K_{1}^{2}+K_{2}^{2}\right)}{K_{1}^{4}\left(K_{1}^{2}+K_{3}^{2}\right)}$$

in which $K_{2}$ and $K_{3}$, are constants assuming the values of 0.1465 and 0.8168 rad/m, respectively; A, is a parameter related to track quality according to the Federal Railroad Administration (FRA), detailed in the authors’ previous work [16]; K, is wave number dependent on the cyclic spatial frequency of irregularity, given as 2π/*λ* and varying between 2π/30 and 2π. More details regarding the track unevenness profile generation are provided by Mosleh et al. [16]. In the numerical simulations, two track profiles shown in Figure 3 are considered: the best “class” according to Federal Railroad Administration (FRA), being “class 6” with A = 37.505 m^3^/rad; and a generated profile from real measurements taken from an inspection vehicle in the Portuguese Northern Line (“class 7” with A = 1.02 m^3^/rad). For reference, a theoretical perfect track profile, nominated as “class 8”, having no irregularities, is also represented.

### 3.5. Train–Track Interaction

The simulations for the train–track dynamic interaction are conducted using the in-house software VSI—Vehicle-Structure Interaction Analysis initially developed by Neves et al. [58] and Montenegro et al. [59], to deal only with vertical interaction, and enhanced by Montenegro et al. [60] to take into consideration the lateral dynamics. The coupling is achieved through a properly validated wheel–rail contact model [60] which is based on a specially developed contact finite element. This element contains a contact formulation divided into three main steps, namely: (i) the geometric contact problem, in which the contact point location between wheel and rail is determined in each step (online approach [61]) based on the solution of a set of nonlinear equations that guarantees the contact compatibility between the two contact surfaces (see [60] for details about the contact surfaces parameterization and the aforementioned geometric nonlinear equations); (ii) the normal contact problem, where the normal forces are computed with the Hertz theory [62]; and (iii) the tangential contact problem, which consists of calculating the creep forces that arise in the contact interface due to the rolling friction contact between wheel and rail using the USETAB routine [63], in which the longitudinal and lateral tangential contact forces are precalculated and stored in a lookup table that can be interpolated during the dynamic analysis as function of the creepages (relative velocities between wheel and rail at the contact point) and the semi axes ratio of the Hertz contact ellipse.

**Figure 4:**
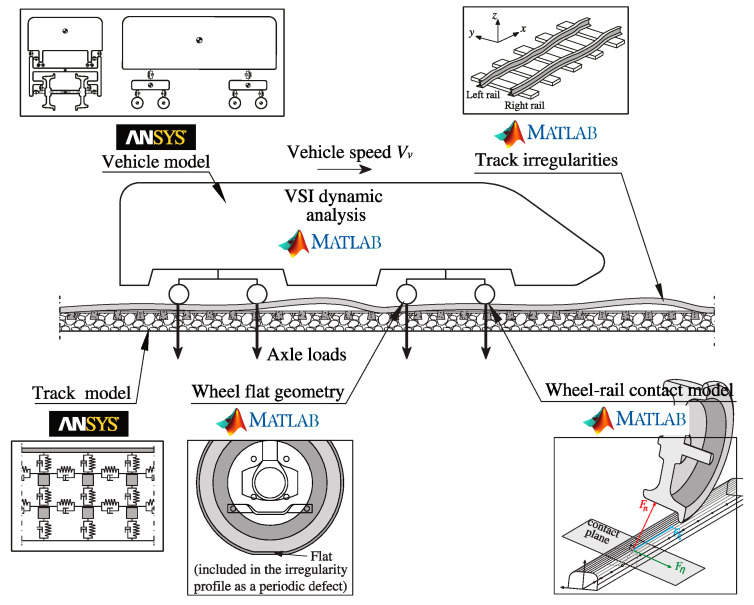
Graphical representation of the numerical modelling. Adapted from [33].

Regarding the dynamic analysis solver, the governing equilibrium equations of both systems are complemented with additional constraint equations that relate the displacements of the contact nodes of the vehicle with the corresponding nodal displacements of the track structure. These equations form a single system, with displacements and contact forces as unknowns, that is solved directly using an optimized block factorization algorithm (see [60] for details). Since this numerical tool is based on the finite element method, it is possible to model structures and vehicles with any degree of complexity. MATLAB^®^ (Release R2018a) [64] implements the numerical tool, which imports structural matrix data from vehicles and tracks previously modelled by ANSYS^®^ (Version 19.2) [65]. present formulation is implemented in MATLAB^®^ (Release R2018a) [64], and the vehicles and structure modeled with ANSYS^®^ (Version 19.2) [65], as schematized in Figure 4. A more detailed description of the train–track interaction tool and wheel–rail contact model, as well as their validation, can be found in Montenegro et al. [60].

The track and the train are modelled in the software ANSYS^®^ [65] as finite element (FE) packages. For the track, beam elements are used to model the rail and sleepers, while flexible behavior of layers such as the ballast and fastening systems are simulated using spring–dashpot elements, and mass point elements are implemented for the mass of the ballast. The track irregularities and the geometry of the wheel flat are introduced in the system through MATLAB^®^ [64]. The train is rendered as a multibody system, with spring–dashpot elements to simulate the flexible behavior of both the primary and secondary suspensions, rigid beams for the rigid body movements of the vehicle, and mass point elements generated at the center of gravity of the carbody, bogies, and wheelsets, to render their mass and inertial effects.

## 4. Wheel Defect Detection: Results and Discussion

The analyses in this study involve a passage of one vehicle of an *Alfa Pendular* train (a typical Portuguese passenger train) circulating through the virtual installed system at different speeds. Only the first vehicle’s right wheel is considered a defective wheel. The virtual accelerometers and SGs are located along a stretch of 2.70 m to monitor the complete perimeter of the wheel.

The analyses are divided into two groups: (i) analyses for wheel flat detection and (ii) analyses for polygonized wheel detection. For each wheel defect, two sensor installation layouts (strain gauges and accelerometers, as presented in Figure 2) are considered to check the sensitivity of the layout scheme on wheel defect detection. Moreover, more sensitivity analyses regarding the train speed, irregularity profile of the rail and wheel defect profiles are conducted.

**Figure 5:**
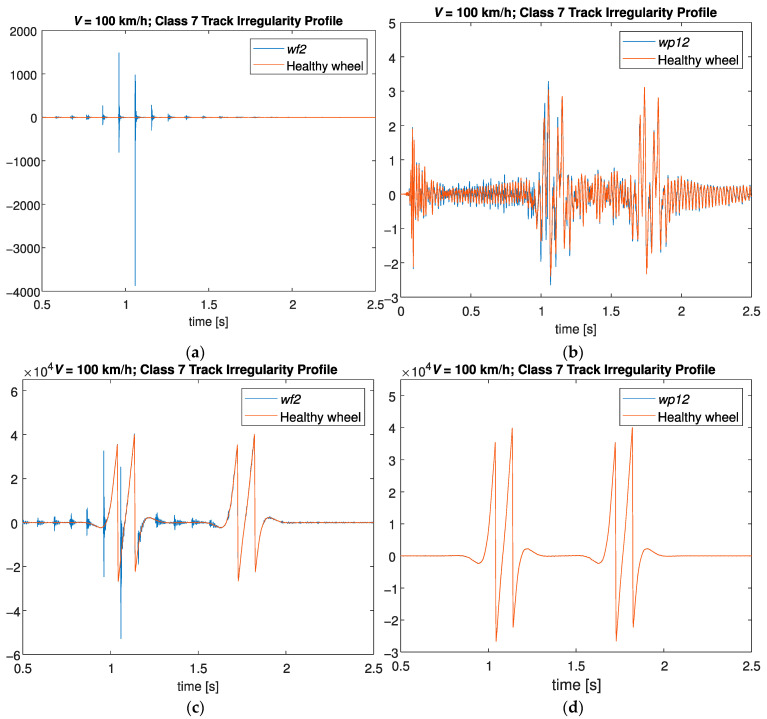
Time-series signal evaluated from position 1: (**a**) acceleration signal for wheel flat profile *wf2* and healthy wheel; (**b**) acceleration signal for polygonized wheel profile *wp12* and healthy wheel; (**c**) shear signal for wheel flat profile *wf2* and healthy wheel; and (**d**) shear signal for polygonized wheel profile *wp12* and healthy wheel.

Figure 5 shows the acceleration and shear time-series signal evaluated from position 1 for both defective and healthy wheels. As presented in these figures, a damaged wheel is not detected by the time domain signal, especially in the presence of a polygonal wheel. Therefore, the main objective of this research is to identify defects on wheels even when they are small, as they may be misinterpreted by operational effects such as changes in train speed.

**Figure 6:**
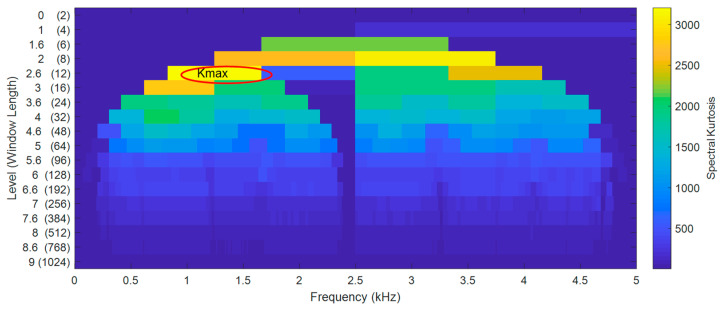
Spectral kurtosis for a shear signal corresponding to the Alfa Pendular train, with maximum kurtosis displayed in the red circle.

By using the kurtosis framework presented previously, the kurtogram for a shear signal corresponding to the passage of the Alfa Pendular train is obtained and presented in Figure 6. The maximum kurtosis is obtained as 3204.5107 at level 2.6, corresponding to the band frequency [$\frac{1}{12}\;\frac{1}{6}$]. With this, the centre and bandwidth frequencies for the corresponding signal, considering Equations (2) and (3), can be determined.

(12) $$\Delta f=2^{\left(-2.6-1\right)}*10,000=824.69\;\mathrm{Hz}$$

$$f_{0}=\left(1+0.5\right)*824.69=1237.03\;\mathrm{Hz}$$

in which $F_{s}=10,000\;\mathrm{Hz}$ is the sampling frequency; Δf=824.69 Hz is the bandwidth frequency; $f_{0}=1237.03\;\mathrm{Hz}$ is the centre frequency.

### 4.1. Wheel Flat Detection by Accelerometers and Strain Gauge Measurements

The variables studied for wheel flat detection are: (i) train speed, (ii) wheel flat geometry, and (iii) track profile irregularity. The track irregularity profiles used for the analyses correspond to “classes 6 and 7”. The train speeds are 100 km/h and 140 km/h.

#### 4.1.1. Sensitivity of Layout Scheme to the Train Speed and Track Irregularity Profile

**Figure 7:**
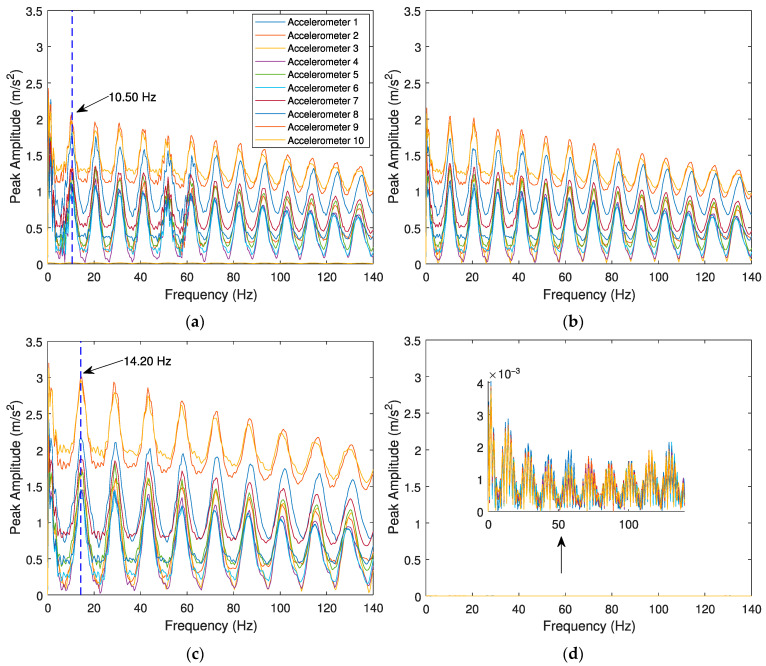
Envelope spectrum analyses obtained from the 10 accelerometers for the Alfa Pendular, considering (**a**) train speed *V* = 100 km/h, “class 6” track irregularity profile and *wf1* wheel flat profile; (**b**) train speed *V* = 100 km/h, “class 7” track irregularity profile and *wf1* wheel flat profile; (**c**) train speed *V* = 140 km/h, “class 7” track irregularity profile and *wf1* wheel flat profile; and (**d**) control scenario with train speed *V* = 140 km/h, “class 7” track irregularity profile and healthy wheel.

**Figure 8:**
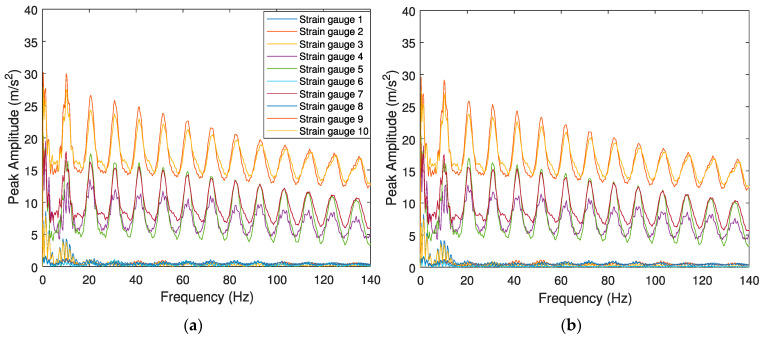

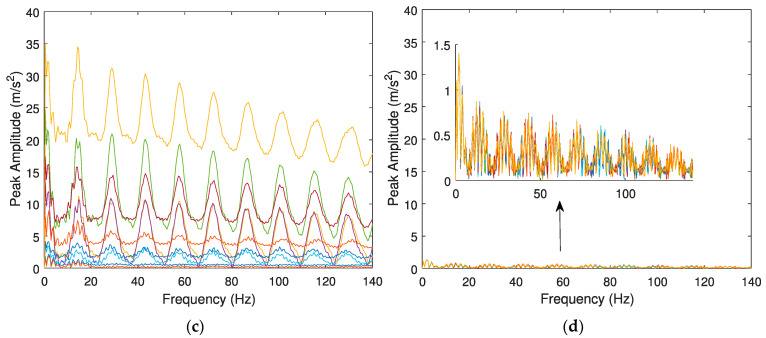
Envelope spectrum analyses obtained from the 10 SGs for the Alfa Pendular, considering (**a**) train speed *V* = 100 km/h, “class 6” track irregularity profile and *wf1* wheel flat profile; (**b**) train speed *V* = 100 km/h, “class 7” track irregularity profile and *wf1* wheel flat profile; (**c**) train speed *V* = 140 km/h, “class 7” track irregularity profile and *wf1* wheel flat profile; and (**d**) control scenario with train speed *V* = 140 km/h, “class 7” track irregularity profile and healthy wheel.

The evaluation of the effects caused by the train speed and irregularity profile of the rail for detecting a wheel flat are conducted with three case scenarios: (i) a train travelling with a speed *V* = 100 km/h on a track section with a “class 6” irregularity profile and a defective wheel with a flat profile *wf1*; (ii) train travelling with a speed *V* = 100 km/h on a track section with a “class 7” irregularity profile and a defective wheel with a flat profile *wf1*; (iii) train travelling with a speed *V* = 140 km/h on a track section with a “class 7” irregularity profile and a defective wheel with a flat profile *wf1*. There is also a control scenario, modelled with a healthy wheel for comparison, considering the smooth track irregularity profile. The envelope spectrum of the signal is presented in Figure 7 and Figure 8 for the response evaluated by accelerometers and SGs, respectively. In the following figures, the vertical dash lines present the wheel flat frequency obtained by Equation (6).

As mentioned before, there are two indicators to distinguish a healthy wheel from a defective one. The first one is the significant lag between signals in the presence of a wheel flat. As shown in the above figures, when a defective wheel passes through the sensors, a notable lag between the envelope spectrum amplitudes is considered. However, all envelope spectrum amplitude for a healthy wheel is coincident (Figure 7d and Figure 8d). Another indicator that demonstrates the presence of a wheel flat is the amplitude variation of the envelope spectral signal, as the amplitude variation of the envelope spectral signal with a wheel flat is higher than a healthy one. These amplitude differences are pronounced between a healthy wheel (Figure 7d and Figure 8d) and a defective one. According to Equation (6), for a train speed of 100 and 140 km/h, the flat impact frequency is calculated as 10.28 and 14.4 Hz. Comparing the theoretical values of flat impact frequency to the ones obtained from the analyses (10.50 and 14.20 Hz indicated in Figure 7a,c), it can be concluded that these values are very similar and confirm the accuracy of the technique.

Figure 7 and Figure 8 present the envelope spectrum for the accelerometers and strain gauges considering the effect of different speeds (*V* = 100 km/h and *V* = 140 km/h), different unevenness profiles of the rail (“classes 6 and 7”), and one type of wheel flat profile (*wf1*, *L* = 20 mm). The effects of track irregularity profiles do not significantly influence the wheel flat detection, as the amplitude differences between several track irregularity profiles (“classes 6 and 7”) for the same train speed *V* = 100 km/h (Figure 7a,b for accelerometers and Figure 8a,b for SGs) are insignificant. In contrast, train speed has an influence on the responses of the envelope spectrum evaluated by accelerometers and SGs, as higher speeds lead to superior peak amplitude values, as shown in Figure 7c and Figure 8c, respectively. The responses of the envelope spectrum for the accelerometers and strain gauges considering a wheel flat (*L* = 20 mm) present a significant lag between signals evaluated by several sensors. However, when a healthy wheel is present (Figure 7d and Figure 8d), the lag between signals is insignificant.

#### 4.1.2. Sensitivity of Layout Scheme to the Wheel Flat Profiles

**Figure 9:**
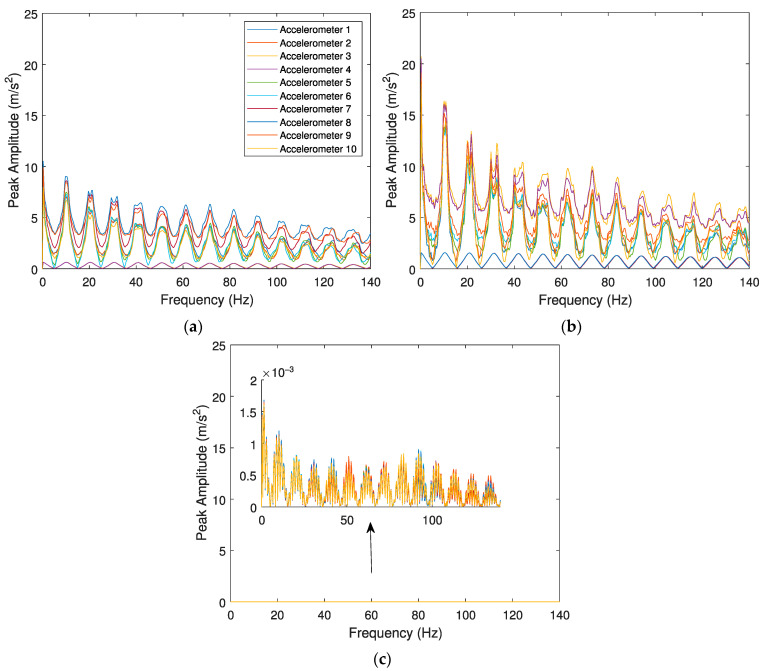
Envelope spectrum analyses obtained from the 10 accelerometers for the Alfa Pendular, considering (**a**) train speed *V* = 100 km/h, “class 7” track irregularity profile and *wf2* wheel flat profile, (**b**) train speed *V* = 100 km/h, “class 7” track irregularity profile and *wf3* wheel flat profile, and (**c**) control scenario with train speed *V* = 100 km/h, “class 7” track irregularity profile and healthy wheel.

**Figure 10:**
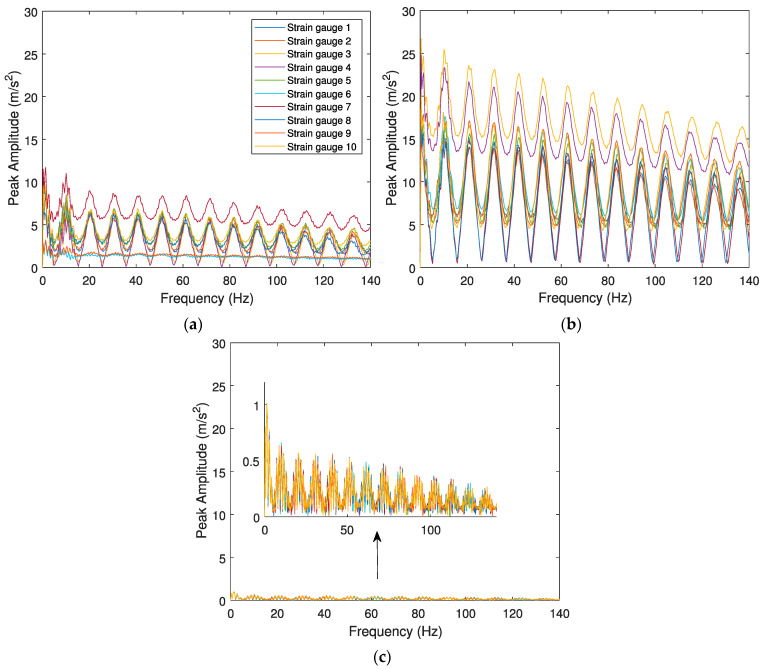
Envelope spectrum analyses obtained from the 10 SGs for the Alfa Pendular, considering (**a**) train speed *V* = 100 km/h, “class 7” track irregularity profile and *wf2* wheel flat profile, (**b**) train speed *V* = 100 km/h, “class 7” track irregularity profile and *wf3* wheel flat profile, and (**c**) control scenario with train speed *V* = 100 km/h, “class 7” track irregularity profile and healthy wheel.

For analyzing the sensitivity of the algorithm to the effects caused by different wheel flat profiles, two case scenarios are considered: the first presenting a wheel flat with a profile *wf2* (*L* = 80 mm), and the second having a wheel flat with a profile *wf3* (*L* = 140 mm). For both scenarios, the train speed is set as 100 km/h, and “class 7” is considered for the rail’s unevenness profile. Similar to the cases presented in Section 4.1.1, a control scenario is presented and modelled with a healthy wheel. The results are presented in Figure 9 and Figure 10 for the responses evaluated by accelerometers and SGs, respectively.

The results show the envelope spectrum obtained from the accelerometers and the SGs, considering different wheel flat profiles. From the results presented above, it can be concluded that the worse wheel flat profiles lead to higher peak amplitude responses. For example, as shown in Figure 7b, for *L* = 20 mm, the maximum amplitude for the envelope spectrum, which is evaluated by acceleration, is around 2 m/s^2^. However, the amplitude of the envelope spectrum in the case of flat length when *L* = 80 mm and *L* = 140 mm, as presented in Figure 9a,b, the amplitude reaches 12 m/s^2^ and 25 m/s^2^, respectively. The envelope spectrum for a healthy wheel indicates a small lag between signals.

### 4.2. Wheel Polygonization Detection from the Accelerometer and Strain Gauge Measurements

The variables studied for wheel polygonization detection are: (i) train speed, (ii) OOR harmonic order, and (iii) unevenness profile of the rail. A polygonized profile with wavelengths between 0.135 and 2.70 m (this being the value of the perimeter of the Alfa Pendular wheel) is considered for simulations. The track irregularity profiles used for the analyses correspond to “classes 6 and 7”. The train speeds are 80 km/h and 140 km/h.

#### 4.2.1. Sensitivity of Layout Scheme to the Train Speed and Track Irregularity Profile

**Figure 11:**
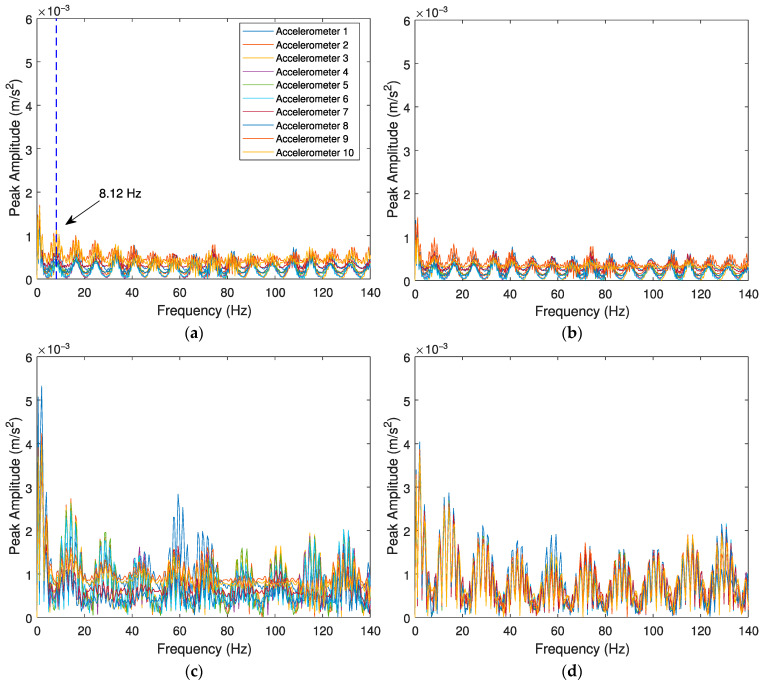
Envelope spectrum analyses obtained from the 10 accelerometers for the Alfa Pendular, considering (**a**) train speed *V* = 80 km/h, “class 6” track irregularity profile and *wp12* polygonized wheel profile, (**b**) train speed *V* = 80 km/h, “class 7” track irregularity profile and *wp12* polygonized wheel profile, (**c**) train speed *V* = 140 km/h, “class 7” track irregularity profile and *wp12* polygonized wheel profile, and (**d**) control scenario with train speed *V* = 140 km/h, “class 7” track irregularity profile and healthy wheel.

**Figure 12:**
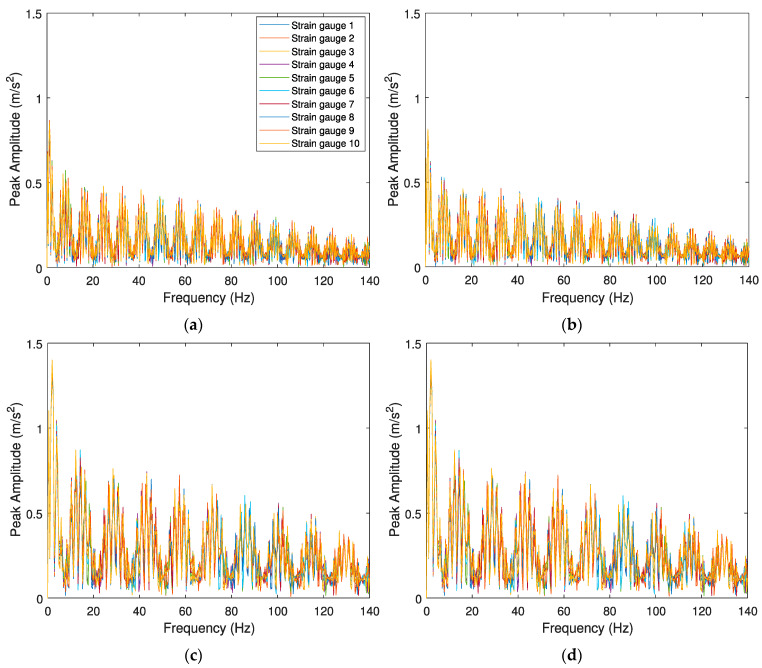
Envelope spectrum analyses obtained from the 10 SGs for the Alfa Pendular, considering (**a**) train speed *V* = 80 km/h, “class 6” track irregularity profile and *wp12* polygonized wheel profile, (**b**) train speed *V* = 80 km/h, “class 7” track irregularity profile and *wp12* polygonized wheel profile, (**c**) train speed *V* = 140 km/h, “class 7” track irregularity profile and *wp12* polygonized wheel profile, and (**d**) control scenario with train speed *V* = 140 km/h, “class 7” track irregularity profile and healthy wheel.

The evaluation of the effects caused by the train speed and irregularity profile of the rail for detecting a polygonized wheel is conducted with three case scenarios: (i) a train travelling with a speed *V* = 80 km/h on a track section with a “class 6” irregularity profile and a defective wheel with a polygonization profile *wp12*; (ii) a train travelling with a speed *V* = 80 km/h on a track section with a “class 7” irregularity profile and a defective wheel with a polygonization profile *wp12*; and (iii) a train travelling with a speed *V* = 140 km/h on a track section with a “class 7” irregularity profile and a defective wheel with a polygonization profile *wp12*. A control scenario with a healthy wheel is also presented. Figure 11 illustrates the envelope spectrum results for accelerometers, and Figure 12 shows the envelope spectrum analyses obtained from positions 1 to 10 (as presented in Figure 2) using SGs.

Figure 11 and Figure 12 present the envelope spectrum analysis for polygonized wheel detection when the signal is evaluated by accelerations and strain gauges, respectively. Once again, the influence of different vehicle speeds (*V* = 80 and *V* = 140 km/h) and two unevenness profiles of the rail (“classes 6 and 7”) are investigated. As mentioned before, there are two indicators to detect a wheel flat: a significant lag between the envelope spectrum amplitudes and the amplitude differences of envelope spectrum analysis between damaged and healthy wheels. However, in the presence of a polygonized wheel, only the lag of the signal as a unique indicator can distinguish a defective wheel from a healthy one. As shown in Figure 11, when a defective wheel passes through the sensors, a lag between the envelope spectrum amplitudes is considered. However, all envelope spectrum amplitude for a healthy wheel is coincident (Figure 11d).

From the results presented in the above figures, it can be inferred that the proposed methodology effectively distinguishes a healthy wheel from a defective one when the signal is evaluated by accelerations. Figure 11 shows the clear lag between signals in the presence of a defect in the wheel, however, all envelope spectrums signals evaluated by SGs (Figure 12) are very similar (no lag), indicating the methodology is not able to detect a polygonized wheel when the signal is evaluated by strain gauges. As shown in Figure 11a,b, although the algorithm can detect a defective wheel considering track irregularities (presented in Figure 3), different roughness of the rail does not lead to the different amplitude of the envelope spectrum. Moreover, by comparing Figure 11b,c, it can be observed that train speed has an influence on the responses of the envelope spectrum evaluated by accelerometers.

#### 4.2.2. Sensitivity of Layout Scheme to the Harmonic Orders

**Figure 13:**
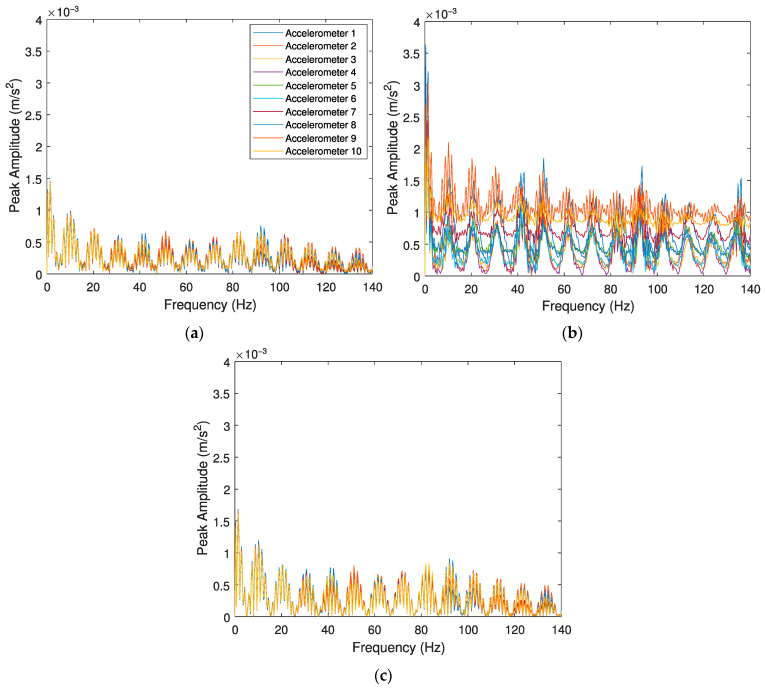
Envelope spectrum analyses obtained from the 10 accelerometers for the Alfa Pendular, considering (**a**) train speed *V* = 100 km/h, “class 7” track irregularity profile and *wp5* polygonized wheel profile, (**b**) train speed *V* = 100 km/h, “class 7” track irregularity profile and *wp20* polygonized wheel profile, and (**c**) control scenario with train speed *V* = 100 km/h, “class 7” track irregularity profile and healthy wheel.

For the algorithm’s sensitivity to the effects caused by different polygonized wheel profiles, two case scenarios are considered: the first presenting a polygonized wheel flat with a profile *wp5*, and the second having a polygonized wheel with a profile *wp20*. Both scenarios feature a train speed *V* = 100 km/h on a track section with a “class 7” irregularity. Like all previous case analyses, a control scenario with a healthy wheel is represented. Given that, in the presence of a polygonized wheel the algorithm cannot distinguish a defective wheel from a healthy one when the signal is evaluated by SGs, only the results of the envelope spectrum assessed by the accelerometers are presented in Figure 13. The results show that as the harmonic orders increase from 5 to 20 (Figure 13a,b), the lag of the envelope spectrum of the signals enhances, indicating the simplicity of detecting the defective wheel.

## 5. Conclusions

This paper aims to detect railway vehicle wheel out-of-roundness using an envelope spectrum analysis. First, a brief explanation of railway vehicle wheel problems is presented, focusing particularly on wheel flats and polygonal wheels. Then, sensitivity analyses are conducted on the type of sensors, unevenness profile of the rail, train speed, as well as different wheel defect profiles. The algorithm simulates the dynamic responses of several strain gauges and accelerometer sensors located between the sleepers. Only the right wheel of the first wheelset is modelled as a defective wheel, but the model allows for various defective wheels in all possible locations. The main achievements from this study are summarized as follows:

- The envelope spectrum methodology is effective in detecting a defective wheel regardless of train speed, rail unevenness profiles, defect type, and amplitude. To perform envelope spectrum analysis, the impulsive signal can be extracted by considering high kurtosis values.
- There are two indicators to detect a wheel flat: a significant lag between the envelope spectrum amplitudes in the presence of flat and the amplitude differences of envelope spectrum analysis between damaged healthy wheels. For polygonized wheels, only the lag of the signal as a unique indicator can distinguish a defective wheel from a healthy one.
- The effects of track irregularity profiles do not significantly influence wheel defect detection (flat and polygonized wheels). In contrast, train speed influences the responses of the envelope spectrum evaluated by accelerometers and SGs, as higher speeds lead to superior peak amplitude values.
- In the presence of a wheel flat, the algorithm is capable of detecting a defective wheel considering both types of sensors (accelerometers and strain gauges), however when a polygonal wheel passes through a system, the algorithm is able to detect a damaged wheel when the signal is evaluated by accelerometers.
- The results show that as the harmonic orders increase from 5 to 20, the lag of the envelope spectrum of the signals enhances, indicating the simplicity of detecting the defective wheel.

For the final development of the proposed methodology, deep learning algorithms would be an effective way to solve the defective detection of the wheel with the sensing data or envelope spectrum. Moreover, it is important to highlight that the authors have the objective of testing the proposed methodology in a real case scenario, which will be the topic of a forthcoming publication.

## Figures and Tables

**Table 1:** Previous wheel flat detection methods.

Wheel Flat Detection Research	Work and Conclusions
Parallelogram Mechanism Method by Gao et al. [26]	Wheel flat detection based on the parallelogram mechanism. Wheel flats could be detected by measuring the change in the vertical displacement of the measuring ruler. Laboratory experiments showed the effectiveness of the method.
Vibration-Based Detection using Envelope Analysis by Wang et al. [27]	Wheel flat vibration-based detection for high-speed trains. Envelope spectrum analysis was performed on axle box vibration signal caused by wheel flats. Results demonstrate that envelope analysis can detect and identify wheel flats with high efficiency and reliability.
Wayside Monitoring System using Envelope Spectrum Analysis by Mosleh et al. [32]	Creation of a wayside monitoring system methodology for wheel flat detection using envelope spectrum analysis. The results demonstrate effectiveness in detecting wheel flats.
Wheel Flat Detection with Multiple Records using Spectral Kurtosis Analysis by Mosleh et al. [31]	Implementation of a multisensory layout scheme for detecting wheel flats on passenger and freight trains. Envelope spectrum and spectral kurtosis were conducted to detect wheel flat. Results show that the system is effective in detecting wheel flats regardless of the position of the sensors or the severity of the flat.
Detection and Evaluation of Wheel Tread Images using Convolution Neural Networks by Trilla et al. [28]	Detection of railway wheel tread defects on raster picture data based on convolutional neural networks that locate the damaged areas in the images, estimate the physical size of the shown defects, and assess their type and severity. Results show that around half the current engineering effort dedicated to manually checking the potential issues can be automated with the implementation of this method.
Advanced Acoustic Signal Analysis for Wheel Flat Detection by Komorski et al. [30]	Detection of wheel flats by using acoustic signals to diagnose rail vehicle wheels based on Hilbert transform and spectrum analysis of acoustic signal envelopes. The results were positive for tram wheel analysis and can be complementary to existing trackside diagnostic systems already installed.
Early wheel flat detection: an automatic data-driven wavelet-based approach for railways by Mosleh et al. [44]	Method for unsupervised early damage detection methodology capable of automatically distinguishing a defective wheel from a healthy one. Results show that one sensor can detect a defective wheel automatically, allowing the development of low-cost and easy-to-install monitoring systems.

**Table 2:** Previous polygonized wheel detection methods.

Wheel Polygonization Detection Research	Conclusions
Out-of-Roundness Monitoring using Polyvinylidene Difluoride (PVDF) Sensing Technology by Song et al. [45]	Wheel OOR detection method based on PVDF strain sensor. The sensors measured the rail strain response caused by wheel–rail interaction to detect defective wheel. The methodology presented was verified by finite element method (FEM) simulations.
Out-of-Roundness Detection Method based on Parameter Optimization (POVMD) and Multinuclear Least Squares-Support Vector Machine (LS-SVM) by Fang et al. [46]	Wheel OOR detection based on POVMD and multi-core LS-SVM. Experimental results show the methodology’s effectiveness in decomposing the vibration signal and analyzing the wheel condition according to its characteristics.
Wheel Tread Polygonization Detection Method based on Dynamic Response by Xu et al. [47]	Wheel polygonization detection method based on synchrosqueezed short-time Fourier transform (SSTFT). Numerical results demonstrate that wheel wear on the high-speed train can be determined in time and ensure the validity of inspection data.
Diagnosis Method of Out-of-Round Metro Wheels with Strong Noise by Huang et al. [48]	Our-of-round defect detection method combining kernel principal component analysis (KPCA) and deep belief network (DBN). KPCA-DBN diagnosis was utilized to distinguish wheels with incipient and severe OOR defects. The results show that the mean accuracy of the diagnosis model can reach 0.9136, demonstrating the effectiveness of the methodology.

**Table 3:** Wheel flat properties.

Wheel Flat Profile	Flat Length (*L*) [mm]	Flat Depth (*D*) [mm]
*wf1*	20	0.058
*wf2*	80	0.93
*wf31*	140	2.80

**Table 4:** Polygonized wheel profiles.

Polygonized Wheel Profile	Harmonic Order (*θ*)	Reasoning
*wp5*	5	Wheels with harmonic orders of five have been identified in operational services [55]
*wp12*	12	Intermediate profile
*wp20*	20	Highest harmonic order possible for this wheel circumference, as noted in Equation (7) [38]

## Data Availability

Data unavailable for public sharing due to privacy reasons.

## References

[1] Song Y., Wang Z., Liu Z., Wang R. (2021). A spatial coupling model to study dynamic performance of pantograph-catenary with vehicle-track excitation. Mech. Syst. Signal. Process.

[2] Pombo J., Ambrósio J. (2013). Environmental and track perturbations on multiple pantograph interaction with catenaries in high-speed trains. Comput. Struct..

[3] Luo R. (2008). Anti-sliding control simulation of railway vehicle braking. Chin. Med. J..

[4] Bernal E., Spiryagin M., Cole C. (2019). Onboard Condition Monitoring Sensors, Systems and Techniques for Freight Railway Vehicles: A Review. IEEE Sens. J..

[5] Bosso N., Gugliotta A., Magelli M., Zampieri N. (2019). Monitoring of railway freight vehicles using onboard systems. Procedia Struct. Integr..

[6] Pintão B., Mosleh A., Vale C., Montenegro P., Costa P. (2022). Development and Validation of a Weigh-in-Motion Methodology for Railway Tracks. Sensors.

[7] Pimentel R., Ribeiro D., Matos L., Mosleh A., Calçada R. (2021). Bridge Weigh-in-Motion system for the identification of train loads using fiber-optic technology. Structures.

[8] Bosso N., Gugliotta A., Zampieri N. (2018). Wheel flat detection algorithm for onboard diagnostic. Measurement.

[9] Cavuto A., Martarelli M., Pandarese G., Revel G.M., Tomasini E.P. (2016). Train wheel diagnostics by laser ultrasonics. Measurement.

[10] Amini A., Entezami M., Papaelias M. (2016). Onboard detection of railway axle bearing defects using envelope analysis of high frequency acoustic emission signals. Case Stud. Nondestruct. Test. Eval..

[11] Zhang Z., Entezami M., Stewart E., Roberts C. (2017). Enhanced fault diagnosis of roller bearing elements using a combination of empirical mode decomposition and minimum entropy deconvolution. Proc. Inst. Mech. Eng. Part C J. Mech. Eng. Sci..

[12] Meixedo A., Goncalves A., Calcada R., Gabriel J., Fonseca H., Martins R. Weighing in motion and wheel defect detection of rolling stock. Proceedings of the 2015 3rd Experiment International Conference (exp.at’15).

[13] Amini A., Entezami M., Huang Z., Rowshandel H., Papaelias M. (2016). Wayside detection of faults in railway axle bearings using time spectral kurtosis analysis on high-frequency acoustic emission signals. Adv. Mech. Eng..

[14] Colaço A., Costa P.A., Connolly D.P. (2016). The influence of train properties on railway ground vibrations. Struct. Infrastruct. Eng..

[15] Mosleh A., Meixedo A., Costa P.A., Calçada R. Trackside Monitoring Solution for Weighing in Motion of Rolling Stock. Proceedings of the TESTE2019—2nd Conference on Testing and Experimentations in Civil Engineering.

[16] Mosleh A., Costa P.A., Calçada R. (2020). A new strategy to estimate static loads for the dynamic weighing in motion of railway vehicles. Proc. Inst. Mech. Eng..

[17] Kouroussis G., Kinet D., Moeyaert V., Dupuy J., Caucheteur C. (2016). Railway structure monitoring solutions using fibre Bragg grating sensors. Int. J. Rail Transp..

[18] Alexandrou G., Kouroussis G., Verlinden O. (2016). A comprehensive prediction model for vehicle/track/soil dynamic response due to wheel flats. Proc. Inst. Mech. Eng. Part F J. Rail Rapid Transit.

[19] Mosleh A., Costa P.A., Caçada R. (2019). Development of a Low-Cost Trackside System for Weighing in Motion and Wheel Defects Detection. Int. J. Railw. Res..

[20] (2020). Rail Industry Safety and Standards Board. Wheel Defects: Code of Practice. http://www.rissb.com.au/products/.

[21] Steenbergen M.J. (2008). The role of the contact geometry in wheel–rail impact due to wheel flats: Part II. Veh. Syst. Dyn..

[22] Jin X., Wu Y., Liang S., Wen Z. (2018). Mechanisms and Countermeasures of Out-of-Roundness Wear on Railway Vehicle Wheels. J. Southwest Jiaotong Univ..

[23] Ahlström J., Karlsson B. (1999). Microstructural evaluation and interpretation of the mechanically and thermally affected zone under railway wheel flats. Wear.

[24] Jergéus J., Odenmarck C., Lundén R., Sotkovszki P., Karlsson B., Gullers P. (1999). Full-scale railway wheel flat experiments. Proc. Inst. Mech. Eng. Part F J. Rail Rapid Transit.

[25] Snyder T., Stone D.H., Kristan J. Wheel flat and out-of round formation and growth. Proceedings of the IEEE/ASME Joint Railroad Conference.

[26] Gao R., He Q., Feng Q. (2019). Railway Wheel Flat Detection System Based on a Parallelogram Mechanism. Sensors.

[27] Wang R., Crosbee D., Beven A., Wang Z., Zhen D. (2020). Vibration-Based Detection of Wheel Flat on a High-Speed Train. Smart Innovation, Systems and Technologies.

[28] Trilla A., Bob-Manuel J., Lamoureux B., Vilasis-Cardona X. (2021). Integrated Multiple-Defect Detection and Evaluation of Rail Wheel Tread Images using Convolutional Neural Networks. Int. J. Progn. Health Manag..

[29] Komorski P., Szymański G.M., Nowakowski T., Orczyk M. Application of the wheel-flat detection algorithm using advanced acoustic signal analysis. Proceedings of the 15th International Conference Dynamical Systems—Theory and Applications.

[30] Komorski P., Szymanski G.M., Nowakowski T., Orczyk M. (2021). Advanced acoustic signal analysis used for wheel-flat detection. Lat. Am. J. Solids Struct..

[31] Mosleh A., Montenegro P.A., Costa P.A., Calçada R. (2021). Railway Vehicle Wheel Flat Detection with Multiple Records Using Spectral Kurtosis Analysis. Appl. Sci..

[32] Mosleh A., Montenegro P., Costa P.A., Calçada R. (2020). An approach for wheel flat detection of railway train wheels using envelope spectrum analysis. Struct. Infrastruct. Eng..

[33] Mosleh A., Meixedo A., Ribeiro D., Montenegro P., Calçada R. (2022). Automatic clustering-based approach for train wheels condition monitoring. Int. J. Rail Transp..

[34] Müller R., Diener M. (1995). Verschleißerscheinungen an Radlaufflächen von Eisenbahnfahrzeugen (Wear phenomena on wheel treads of railway vehicles). ZEV DET Glas. Ann. Die Eisenb..

[35] Rode W., Müller D., Villman J. (1997). Results of DB AG investigations “out-of-round wheels”. Corrugation Symposium—Extended Abstracts, IFV Bahntechink.

[36] Morys B. (1999). Enlargement of out-of-round wheel profiles on high speed trains. J. Sound Vib..

[37] Meinke P., Meinke S. (1999). Polygonalization of wheel treads caused by static and dynamic imbalances. J. Sound Vib..

[38] Johansson A., Andersson C. (2005). Out-of-round railway wheels—A study of wheel polygonalization through simulation of three-dimensional wheel–rail interaction and wear. Veh. Syst. Dyn..

[39] Jin X., Wu L., Fang J., Zhong S., Ling L. (2012). An investigation into the mechanism of the polygonal wear of metro train wheels and its effect on the dynamic behaviour of a wheel/rail system. Veh. Syst. Dyn..

[40] Ma W., Song R., Luo S. (2016). Study on the mechanism of the formation of polygon-shaped wheels on subway vehicles. Proc. Inst. Mech. Eng. Part F J. Rail Rapid Transit.

[41] Peng B., Iwnicki S., Shackleton P., Zhao Y., Cui D. (2018). A practical method for simulating the evolution of railway wheel polygonalization. The Dynamics of Vehicles on Roads and Tracks.

[42] Zhao X.N., Chen G.X., Lv J.Z., Zhang S., Wu B.W., Zhu Q. (2019). Study on the mechanism for the wheel polygonal wear of high-speed trains in terms of the frictional self-excited vibration theory. Wear.

[43] Ye Y., Shi D., Krause P., Tian Q., Hecht M. (2020). Wheel flat can cause or exacerbate wheel polygonization. Veh. Syst. Dyn..

[44] Mosleh A., Meixedo A., Ribeiro D., Montenegro P., Calçada R. (2022). Early wheel flat detection: An automatic data-driven wavelet-based approach for railways. Veh. Syst. Dyn..

[45] Song Y., Wang Z., Du Y. (2014). Study on Train Wheel Out-of-Roundness Monitoring Method by PVDF Sensing Technology. Open Mech. Eng. J..

[46] Fang L., Li S., Dai W., Zhang Y., Xing Z., Han Y. (2020). Method of Wheel Out-of-Roundness Detection Based on POVMD and Multinuclear LS-SVM. Lecture Notes in Electrical Engineering.

[47] Xu X., Liu J., Sun S., Xie W. Detection method for polygonalization of wheel treads based on dynamic response. Proceedings of the ACM International Conference Proceeding Series.

[48] Huang H., Wang H., Zhang W., Gu W. (2021). A Fault Diagnosis Method for Out-of-Round Faults of Metro Vehicle Wheels with Strong Noise. Shock. Vib..

[49] Mosleh A., Montenegro P.A., Costa P.A., Calçada R., Calçada R., Kaewunruen S. (2022). Approaches for weigh-in-motion and wheel defect detection of railway vehicles. Rail Infrastructure Resilience, a Best-Practices Handbook. Woodhead Publishing Series in Civil and Structural Engineering.

[50] Silva R., Guedes A., Ribeiro D., Vale C., Meixedo A., Mosleh A., Montenegro P. (2023). Early Identification of Unbalanced Freight Traffic Loads Based on Wayside Monitoring and Artificial Intelligence. Sensors.

[51] Hasan T., Brillinger D.R., Krishnaiah P.R. (1983). Complex demodulation: Some theory and applications. Time Series in the Frequency Domain. Handbook of Statistics.

[52] Antoni J. (2007). Fast computation of the kurtogram for the detection of transient faults. Mech. Syst. Signal Process..

[53] Antoni J. (2006). The spectral kurtosis: A useful tool for characterising non-stationary signals. Mech. Syst. Signal Process..

[54] Zhai W.M., Wang Q.C., Lu Z.W., Wu X.S. (2001). Dynamic effects of vehicles on tracks in the case of raising train speeds. Proc. Inst. Mech. Eng. Part F J. Rail Rapid Transit.

[55] Tao G., Wen Z., Liang X., Ren D., Jin X. (2018). An investigation into the mechanism of the out-of-round wheels of metro train and its mitigation measures. Veh. Syst. Dyn..

[56] Fries R.H., Coffey B.M. (1990). A State-Space Approach to the Synthesis of Random Vertical and Crosslevel Rail Irregularities. J. Dyn. Syst. Meas. Control..

[57] Hamid A., Yang T.L. (1982). Analytical description of track-geometry variations. Transportation Research Record.

[58] Neves S., Montenegro P., Azevedo A., Calçada R. (2014). A direct method for analyzing the nonlinear vehicle–structure interaction. Eng. Struct..

[59] Montenegro P.-A., Neves G.-M., Ferreira M.-A.A., Calçada R. A Nonlinear Vehicle Structure Interaction Methodology with Wheel Rail Detachment and Reattachment. Proceedings of the 4th International Conference on Computational Methods in Structural Dynamics and Earthquake Engineering.

[60] Montenegro P., Neves S., Calçada R., Tanabe M., Sogabe M. (2015). Wheel–rail contact formulation for analyzing the lateral train–structure dynamic interaction. Comput. Struct..

[61] Sugiyama H., Araki K., Suda Y. (2009). On-line and off-line wheel/rail contact algorithm in the analysis of multibody railroad vehicle systems. J. Mech. Sci. Technol..

[62] Hertz H.R. (1882). Ueber die Berührung fester elastischer Körper (On Contact Between Elastic Bodies). J. Für Die Reine Und Angew. Math. (Crelle’s J.).

[63] Kalker J.J. (1996). Book of Tables for the Hertzian Creep-Force Law.

[64] MATLAB^®^ (2018). Version 9.4 (R2018a).

[65] ANSYS^®^ (2018). Academic Research Version 19.2.

